# Contemporary Management and Prognostic Factors of Arrhythmia Recurrence in Patients with High-Energy Discharge of Cardiac Implantable Electronic Devices

**DOI:** 10.3390/medicina60101662

**Published:** 2024-10-10

**Authors:** Zofia Kampka, Mateusz Drabczyk, Magdalena Pająk, Olga Drapacz, Michał Orszulak, Małgorzata Cichoń, Katarzyna Mizia-Stec, Maciej T. Wybraniec

**Affiliations:** First Department of Cardiology, School of Medicine in Katowice, Medical University of Silesia, 47 Ziołowa St., 40-635 Katowice, Poland; zofiakampka@gmail.com (Z.K.); matidra1998@gmail.com (M.D.); magdalenapajak9626@gmail.com (M.P.); olga.drapacz@gmail.com (O.D.); orszul@vp.pl (M.O.); malgorzata.cichon3@gmail.com (M.C.); kmiziastec@gmail.com (K.M.-S.)

**Keywords:** implantable cardioverter-defibrillator, ICD, discharge, coronary angiography, catheter ablation

## Abstract

*Background and Objectives*: Understanding the underlying causes of implantable cardioverter-defibrillator (ICD) discharges is vital for effective management. This study aimed to evaluate the characteristics of patients admitted following ICD discharge, focusing on myocardial ischemia as a potential exacerbating factor and potential risk factors for VT recurrence. *Materials and Methods*: This retrospective, single-center study included 81 patients with high energy discharge from cardiac implantable electronic device admitted urgently to the cardiology department from 2015 to 2022. The exclusion criterion was ST-segment elevation acute coronary syndrome. Data were collected anonymously from electronic medical records. Patients were categorized based on coronary angiography, percutaneous angioplasty, presence of significant stenosis, recurrent ventricular tachycardia (VT), and catheter ablation. Clinical variables, including demographic data, echocardiographic parameters, and pharmacotherapy, were analyzed. The primary endpoint was the recurrence of VT during in-hospital stay. *Results*: Among 81 patients, predominantly male (86.4%), with a mean age of 63.6 years, 55 (67.9%) had coronary artery disease (CAD) as the primary etiology for ICD implantation. Coronary angiography was performed in 34 patients (42.0%) and showed significant stenosis (>50%) in 18 (41.8%) patients, while 8 (26.0%) individuals underwent percutaneous coronary intervention (PCI). Recurrent VT occurred in 21 subjects (26.3%), while ventricular catheter ablation was performed in 36 patients (44.0%). Referral for urgent coronary angiography was associated with presence of diabetes (*p* = 0.028) and hyperlipidemia (*p* = 0.022). Logistic regression analysis confirmed NYHA symptomatic class (OR 4.63, *p* = 0.04) and LVH (OR 10.59, *p* = 0.049) were independently associated with relapse of VT. CAD patients underwent catheter ablation more frequently (*p* = 0.001) than those with dilated cardiomyopathy. *Conclusions*: The study showed a low referral rate for coronary angiography among patients with ICD discharge. Presence of LVH and preexisting symptomatic class influence arrhythmia recurrence. Understanding these associations can guide personalized management strategies for ICD recipients.

## 1. Introduction

The growing number of implantable cardioverter-defibrillator (ICD) recipients results in an increased frequency of ICD-related problems, creating the pressing need to address them [1]. While ICDs undoubtedly contribute to the prevention of sudden cardiac death (SCD) [2], it should be noted that in patients suffering from heart failure (HF), ICD shocks are associated with increased mortality [3].

According to ESC/ACC guidelines, the primary prevention of SCD entails ICD implantation in patients with symptomatic HF and left ventricular ejection fraction (LVEF) ≤ 35% despite ≥ 3 months of best medical therapy [4], while secondary prevention concerns survivors of ventricular fibrillation or hemodynamically unstable ventricular tachycardia [5]. ICD discharges and recurrent arrhythmias are negative prognostic factors; therefore, the identification of possible underlying causes seems to be a vital step in the management of HF patients. Despite the rapid expansion of ICD application [6], the available data concerning the treatment after the occurrence of ICD discharge are very limited. What we know is that ICD discharges/electrical storms may be triggered by ventricular fibrillation or ventricular tachycardia caused by reentrant tachycardia conditioned by myocardial scar in structural heart disease; may result from active myocardial ischemia, hypokalemia, hypoxia, or inflammatory response; or may occur as an inappropriate response to ICD malfunction or supraventricular arrhythmia, such as atrial fibrillation (AF) or flutter [7]. The underlying causes need to be actively searched for and treated.

As coronary artery disease (CAD) represents the main etiology of HF leading to ICD implantation, active ischemia should be considered in all patients following ICD discharge. Additionally, patients with non-ischemic HF etiology may develop CAD overlapping other etiologies of HF. Therefore, coronary angiography should be considered in selected patients; however, it is not directly established in contemporary guidelines [1,8]. Consequently, there is a pressing need to explore the characteristics of patients admitted to the cardiology department due to cardiac implantable device discharge, with a particular emphasis on myocardial ischemia as a potential exacerbating factor in their condition. We also looked for VT recurrence trigger factors in this group of patients. To achieve this goal, the present study sought to comprehensively investigate the demographic, clinical, and prognostic factors associated with patients experiencing ICD discharges. By focusing on the potential role of myocardial ischemia in exacerbating the disease process, we aim to shed light on the relevance of coronary evaluation in this patient population.

## 2. Materials and Methods

This was a retrospective, observational, single-center study that covered 81 patients admitted to the First Department of Cardiology, Medical University of Silesia, Upper-Silesian Medical Center in Katowice, Poland, from 2015 to 2022. The data were obtained from electronic medical records and gathered anonymously. The study group embraced patients who were urgently admitted to the hospital due to high-energy ICD/CRT-D discharge triggered by ventricular tachycardia (VT), excluding patients with STEMI-related VT.

The analyzed parameters taken into consideration comprised sex, age, body mass index (BMI), and past medical history including the number of PCI (percutaneous coronary intervention) or CABG (coronary artery bypass grafting) procedures before the aforementioned urgent admission. We also collected information on the past diagnoses of myocardial infarction/unstable and stable angina, concomitant disease indication for cardiac implantable electronic device implantation, details of the high-energy interventions, baseline parameters obtained using transthoracic echocardiography (TTE), electrocardiogram (ECG) or Holter ECG monitoring, procedural characteristics of the percutaneous coronary intervention (PCI) and ablation procedures, pharmacotherapy, blood test results, and family history.

The primary clinical endpoint was the recurrence of ventricular tachycardia (VT) during hospital observation.

The decision to perform coronary angiography was based on an individual case-based approach.

Significant coronary artery stenosis was defined as >50% diameter stenosis based on angiography. Electrical storm was defined as at least ≥3 appropriate high energy ICD/CRT-D discharges within 24 h.

The study was carried out in accordance with the Declaration of Helsinki and the study protocol was accepted by the Ethics Committee of the Medical University of Silesia in Katowice.

### Statistical Analysis

Statistical analysis was performed using SPSS v.25.0 software (IBM Corp, Armonk, NY, USA). Shapiro–Wilk’s test was used to verify distribution of continuous variables, which are presented as arithmetic mean value ± standard deviation (SD) or median. Categorical variables are expressed as absolute counts with percentages (%). The Mann–Whitney test or Wilcoxon W (Kruskal–Wallis) test was applied in the case of continuous variables, while qualitative parameters were utilized by the χ^2^ test (Pearson chi-Square tests). In univariate analysis, odds ratio (OR) and 95% confidence intervals (CI) were calculated to establish predictors of outcome. Stepwise logistic regression analysis incorporated all variables with *p* < 0.1 in univariate analysis. A ‘*p*’ value of <0.05 was regarded as statistically significant throughout the study analyses.

## 3. Results

**Figure 1:**
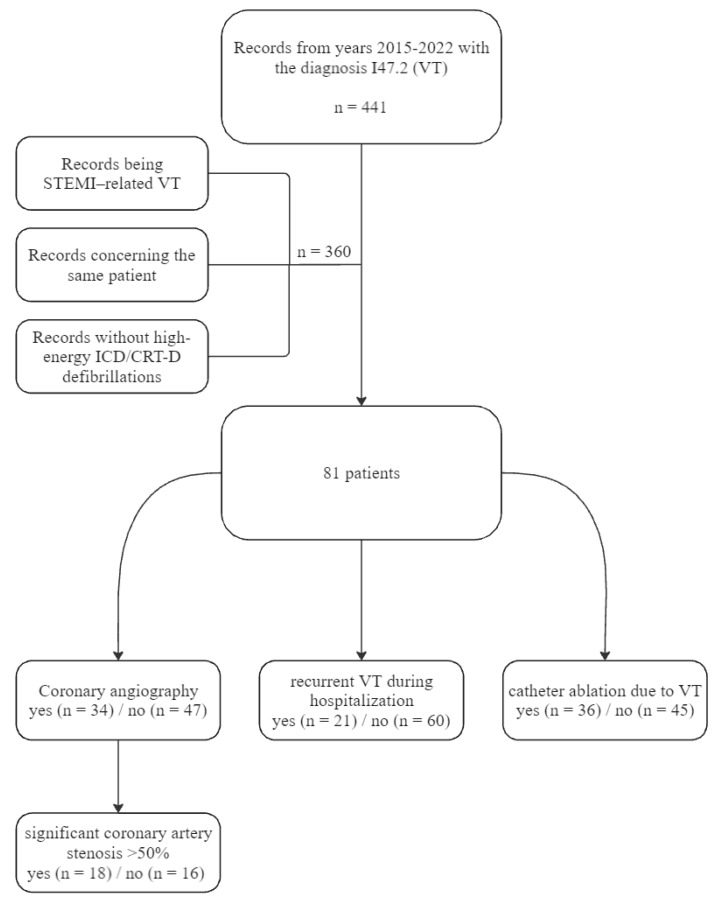
A flow chart with patient selection details.

During the selection process, we initially searched the database for patients with the diagnosis of VT using the International Classification of Diseases (ICD) billing codes (10th edition, I47.2). The primary inclusion criteria for the study were a history of high energy discharge from ICD or cardiac resynchronization therapy—defibrillator (CRT-D) as the reason for the hospital admission. The major exclusion criterion was ST-segment myocardial infarction as a cause of ICD intervention. The medical records which were missing such essential data as primary endpoint were excluded. This eventually led to selection of 81 patients, who were subsequently divided into several groups, based on whether they underwent coronary angiography (n = 34) or not (n = 47), had recurrent VT during hospitalization (n = 21) or not (n = 60), and underwent catheter ablation due to VT (n = 36) or not (n = 45). Furthermore, in the study group of 34 patients who underwent coronary angiography, we delineated a subset of patients who had significant coronary artery stenosis >50% (n = 18) or not (n = 16). The simplified selection process is presented in a flow chart (Figure 1)**.** These mentioned groups were compared in the final analysis.

**Figure 2:**
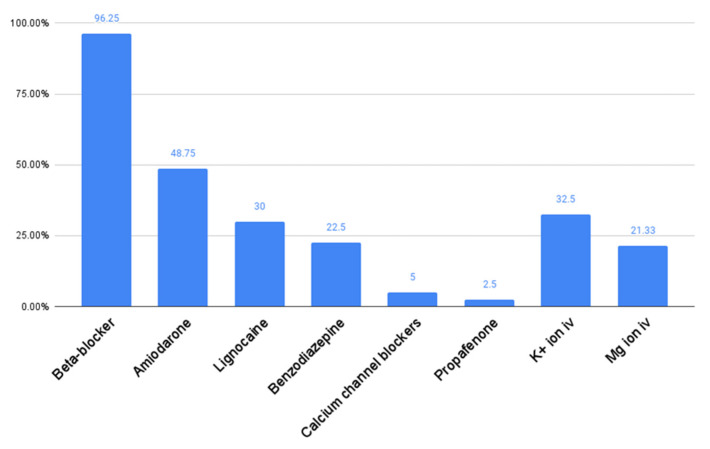
In-hospital pharmacotherapy received by patients.

A total of 81 patients were included in the study, with a mean age of 63.6 ± 12.6 years, weight of 86.6 ± 21.6 kg, and height of 170.9 ± 13.2 cm. The study population consisted of 70 (86.4%) men and 11 (13.6%) women, with a mean BMI of 28.4 ± 5.2 kg/m², left ventricular ejection fraction (LVEF) of 29.5 ± 12.4%, and left atrial diameter (LAd) of 46.0 ± 7.2 mm. Among the analyzed cohorts, 57 (70.4%) patients had an implanted ICD, while CRT was implanted in 24 (29.6%) individuals. The etiology of pacing device implantation was coronary artery disease (CAD) in 55 (67.9%) patients and dilated cardiomyopathy (DCM) in 26 (32.1%) recipients. Prior to hospital admission, beta-blockers were administered in 75 patients (96.2%), calcium channel blockers in 5 patients (6.5%), amiodarone in 32 patients (43.2%), and propafenone in 2 patients (2.6%). During hospitalization, beta-blockers were applied in 77 patients (96.2%), calcium channel blockers in 4 patients (5.0%), amiodarone in 39 patients (48.8%), propafenone in 2 patients (2.5%), lignocaine in 24 patients (30.0%), benzodiazepines in 18 patients (22.5%), intravenous potassium supplementation in 25 patients (32.5%), and iv magnesium in 16 patients (21.3%). The received in-hospital pharmacotherapy is presented in Figure 2. Further demographic and clinical characteristics of the study group are summarized in Table 1.

### 3.1. Coronary Angiography and Presence of Coronary Artery Stenosis >50%

Coronary angiography was performed in 34 patients (42.0% of overall population), while stenosis >50% in epicardial coronary arteries was documented in 18 patients (52.9%) and only 8 patients underwent PCI (26.0% of patients managed invasively). The comparison of clinical variables between the groups of patients who underwent coronary angiography and who did not is shown in Table 1. Patients who were referred for urgent coronary angiography had mainly ischemic HF as an indication for ICD implantation (88.2%, *p* = 0.001). Moreover, in this study group, atrial fibrillation (AF) was less frequently noted during ECG performed on admission (*p* = 0.042), although there were no statistically significant differences in AF prevalence in past medical history. They were more often diagnosed with hyperlipidemia (*p* = 0.022) and diabetes (*p* = 0.028), and had a history of myocardial infarction (MI) (*p* = 0.016) and PCI or both PCI and coronary artery bypass grafting (CABG) in the past (*p* < 0.001). There was a trend towards a higher level of maximal troponin in patients referred for coronary angiography in comparison to patients managed conservatively (0.044 ± 0.04 vs. 0.162 ng/mL ± 0.2, *p* = 0.093). Furthermore, patients who underwent coronary angiography were more often treated with lignocaine (*p* = 0.018) and intravenous potassium (*p* = 0.019) during the hospitalization; however, the frequency of amiodarone administration remained statistically insignificant.

The analysis of clinical variables between the individuals with and without coronary artery stenosis >50% demonstrated that patients with stenosis >50% had a trend towards smaller left atrial diameter on transthoracic echocardiography (TTE) (43.9 ± 5.4 vs. 47.9 ± 6.0, *p* = 0.058) and a trend for greater prevalence of intraventricular conduction delay (33.3% vs. 0%, *p* = 0.058), hyperthyroidism (33.3% vs. 6.3%, *p* = 0.051) in medical history, and a higher level of serum potassium (4.6 ± 0.5 vs. 4.2 ± 0.4, *p* = 0.050).

Additionally, they were more frequently administered amiodarone (*p* = 0.049) and benzodiazepines (*p* = 0.005). Of note, the level of troponin (0.116 vs. 0.042 ng/mL, *p* = 0.704), and prevalence of LBBB (22.2% vs. 8.3%, *p* = 0.368) and RBBB (22.2% vs. 25.0%, *p* = 0.882), were comparable in both groups. Still, none of the presented variables was an independent predictor of the presence of significant stenosis in coronary angiography.

### 3.2. Recurrent VT during Hospitalization

The analysis of different variables depending on the relapse of VT during hospitalization is presented in Table 2. The analysis showed that patients who experienced recurrent VT during hospitalization had higher New York Heart Association class (NYHA; 2.2 ± 0.6 vs. 2.9 ± 0.8, *p* = 0.028), a higher number of PVCs (*p* = 0.012), and minimal heart rate in Holter monitoring (*p* = 0.033), as well as higher WBC (*p* = 0.018) and serum creatinine concentration (*p* = 0.007) levels, along with a lower eGFR level (*p* = 0.002) and greater QTc (*p* = 0.028). Furthermore, patients with recurrent VT more often experienced ICD intervention (*p* < 0.001) and reintervention after standard therapy (*p* < 0.001), as well as underwent catheter ablation (*p* < 0.001) during in-hospital stay. Patients with VT recurrence had greater prevalence of LVH (*p* = 0.006), chronic kidney disease (*p* = 0.028), and intensive insulin therapy (*p* = 0.013). Moreover, patients with VT relapse were more often in the course of chronic amiodarone (*p* = 0.042) and statin treatment (*p* = 0.045) before hospitalization, whereas they also more often received amiodarone (*p* = 0.007), benzodiazepines (*p* = 0.030), and magnesium (*p* = 0.023) during hospitalization.

The results of univariate and logistic regression analyses of different predictors of recurrent VT during hospitalization are summarized in Table 3. Logistic regression analysis demonstrated that chronic symptomatic NYHA class (unit OR 4.63, *p* = 0.04) and presence of left ventricular hypertrophy on TTE (OR 10.59, *p* = 0.049) are independently associated with recurrent VT (area under the receiver operating characteristic curve (AUROC) = 0.853, 95%CI 0.716 to 0.941; Hosmer–Lemeshow *p* = 0.57).

### 3.3. Catheter Ablation Due to VT during Hospitalization

The analysis unveiled that in patients submitted to catheter ablation, the etiology of ICD implantation was mostly CAD (*p* = 0.001) and the prevalence of electrical storms was significantly higher (59.5% vs. 84.8%, *p* = 0.017). Of note, an increased incidence of persistent VT (*p* = 0.007) and ICD interventions during hospitalization (*p* < 0.001) in this group was observed. Furthermore, patients undergoing VT ablation had significantly lower EF (*p* = 0.049) and more prevalent history of MI (*p* = 0.019), were more often treated with statins (*p* = 0.022), and more frequently received benzodiazepines (*p* < 0.001) and lignocaine (*p* = 0.039) during in-hospital stay. Moreover, patients in this group had a greater level of white blood cells (WBCs) (*p* = 0.016), potassium (*p* < 0.001), triglycerides (*p* = 0.034), thyroid stimulating hormone (TSH) (*p* = 0.011), and creatinine (*p* = 0.012), and a lower level of eGFR (*p* = 0.020).

## 4. Discussion

This retrospective study aimed to evaluate the predictors of recurrent VT during hospitalization in patients with discharge of a cardiac implantable electronic device, with a special focus on referral for coronary angiography and the presence of significant CAD. Specifically, we sought to determine the necessity of coronary angiography for all patients admitted to the hospital following high-energy ICD interventions. The main finding of our study is that roughly 42% of patients with ICD/CRT-D discharge are subject to urgent coronary angiography, while stenoses >50% in epicardial coronary arteries are present in only 52.9% of them. The presence of significant CAD and PCI during index hospitalization did not predict recurrent VT during in-hospital stay, while the presence of left ventricular hypertrophy and prior NYHA symptomatic class are associated with relapse of ventricular arrhythmia. These results are in line with a study by Chatterjee et al., which found that patients with LVH had 2.8 times higher odds of experiencing VT and VF compared to those without LVH [9]. The diagnosis of hypertrophic cardiomyopathy (HCM) is a well-established risk factor of sudden cardiac death, prompting the need for ICD implantation in primary prevention [10]. In our population, only seven patients (8.6%) were characterized by HCM diagnosis, while the majority of patients had primarily hypertension-mediated LVH, which still corresponded with the risk of recurrence of ventricular arrhythmia (Table 3).

It is important to note that neither pre-hospital NSVT nor PVC burden was associated with recurrent VT during hospitalization. Likewise, the number of PVCs was not significant statistically. According to Rune Boas et al., NSVT and high burden of PVC were both associated with increased all-cause mortality and CVD, although there was no statistically significant association with SCD for either high burden of PVC or NSVT [11].

In our study, higher minimal heart rate in Holter monitoring was present in the group of patients with VT recurrence. Kouakam et al. found that elevated heart rate preceding the onset of VT was associated with unsuccessful conversion without ICD discharge, suggesting sympathetic activation as the underlying cause. Shortened refractoriness and conduction caused by sympathetic activation have a proarrhythmic character, stressing the need to use beta-blockers in those patients [12].

While our study does not include data on the rate of VT, the authors would like to stress its clinical significance. Although slow VT, defined as VT <150 bpm and cycle length >320 ms, is commonly present in ICD recipients and may concern even one-third of patients, it does not seem to be of a wide clinical relevance. Most of these episodes are short-lasting, scarcely symptomatic or asymptomatic, not leading to a life-threatening situation, and efficiently terminated by ATP [13]. On the other hand, very fast VT (cycle length 200–250 ms) is a clinically significant arrhythmia, which can be successfully terminated not only by high-energy shocks, but also by ATP and low-energy cardioversion. However, the success rate of ATP is higher in fast VT (251–320 ms) than in very fast VT [14].

Based on the ESC guidelines, potential treatment options following ICD shocks include antiarrhythmic therapy with amiodarone or the use of ablation [8]. Kleemann et al. suggested a three-step management concept in ICD discharge patients summarized as the ToVAMI therapy, which relates to Trigger identification and optimization (To), Ventricular Arrhythmia (VA) treatment, and Medical and Interventional (MI) prognostic heart failure treatment. Additionally, to facilitate the trigger identification, they introduce the acronym ICD-STEMi, which stands for Ischemia, Compliance, Decompensation, Stress, Technical, Electrolyte/endocrinologic disorders, and Medication intoxication [15]. Our study stands in line with Kleemann et al., who suggested that the adequate therapy after ICD shock is far more complex than amiodarone administration or the ablation procedure presented in guidelines.

Our research revealed a strong association between renal function and susceptibility to ventricular tachycardia. The group of patients who experienced recurrent VT during the stay at the hospital had significantly lower eGFR levels, a higher creatinine level, and significantly more often suffered from chronic kidney disease. These findings are consistent with the study of Weidner K. et al., where the presence of chronic kidney disease was associated with a higher risk of recurrent ventricular tachyarrhythmias [16]. Research trials indicate that the most common causes of arrhythmia, including VT in chronic renal disease, include electrolyte disturbances, uremia, and hemodialysis-induced hemodynamic stress [17]. Additionally, the incidence of sudden cardiac death, triggered by ventricular arrhythmia, is the most common cause of death in hemodialyzed patients and accounts for one-quarter of all-cause mortality [18]. Therefore, patients suffering from chronic kidney disease should be surrounded with special care, being carefully watched for reversible trigger factors for VT such as electrolyte disturbances [8].

Our study revealed a significant correlation between the use of intensive insulin therapy and occurrence of new VT episodes. Andersen et al. reported that in insulin-treated patients, hypoglycemia causes clinically significant increases in cardiac repolarization, which can lead to a higher level of vulnerability for ventricular arrhythmias and sudden cardiac death [19,20]. Consequently, patients with a high risk of hypoglycemia, especially those treated with insulin, should have their glycemic levels closely monitored [21].

Based on our findings, individuals with elevated WBC levels exhibited a higher susceptibility to recurrent VT during hospitalization. Additionally, we observed a prominent discrepancy between the CRP mean level of groups of patients with and without experiencing recurrent VT during their stay at the hospital (26.0 ± 18.6 vs. 75.4 ± 139.7). This may suggest that inflammation could potentially compromise the clinical effectiveness of the treatment in this group of patients. Increased levels of inflammatory markers have already been associated with severe VT in the literature [22], although, on the contrary, several clinical trials revealed that the CRP level cannot be used to predict VT [23,24].

In our study, PCI was performed in as low as eight patients (9.9% of overall population), and myocardial revascularization was not a predictor of VT recurrence. It is well known that ongoing myocardial ischemia is a potent proarrhythmic factor that translates into risk of first and recurrent episodes of sustained VT [25]. Still, based on the results of the recent TOMAHAWK randomized controlled trial performed in patients with out-of-hospital cardiac arrest, coronary angiography should be postponed if the initial ECG does not reveal ST-segment elevations [26]. Ischemia is thus one of many possible contributors to the onset of VT and ventricular fibrillation, along with inflammation, infection, hypokalemia, hypomagnesemia, hyperthyroidism, and elevated QTc related with drug use or alcohol abuse [27].

According to our study, patients with diabetes and hyperlipidemia were more likely to undergo coronary angiography during hospitalization. This falls in line with well-established knowledge of coronary artery disease risk factors [28]. These individuals were also more frequently treated with ASA and statins, fundamental medications included in most guidelines for management of the mentioned condition [29,30]. Determining the accurate medical history may be necessary to establish further steps of a treatment plan in the patients.

In our investigation, patients with coronary artery disease (CAD) underwent catheter ablation more frequently than those with dilated cardiomyopathy (DCM). This suggests a potential predisposition for ventricular arrhythmias among CAD patients. However, Streitner et al. did not identify any significant disparity in the occurrence of ventricular tachycardia (VT) and ventricular fibrillation (VF) between these populations [31]. Interestingly, Dinov et al. observed that the long-term outcomes of VT ablation were less favorable in patients with DCM compared to those with ischemic changes [32]. Notably, ablation should be considered because of lowering the odds of VT storm and cardiac hospitalizations in CAD patients [33]. Patients with history of MI and VT refractory to antiarrhythmic drugs can especially benefit from this procedure [34].

In addition, our research reveals that individuals who have undergone catheter ablation exhibit a notable decrease in ejection fraction (EF) alongside an increase in end-systolic volume (ESV). These observed changes suggest a heightened susceptibility to arrhythmias within this group. Notably, Di Bella et al.’s study underscores the significance of ESV in predicting ventricular tachycardia (VT), particularly in cases of non-sustained tachycardia, as determined by multivariate analysis [35].

Over 95% of patients in our study were administered beta-blockers in the pre-hospital and in-hospital pharmacotherapy. Higher minimal HR in Holter monitoring was associated with higher chance of VT recurrence, with maximal HR being of no account; however, this is not reflected in other studies. Sun X. et al. found a U-shaped association between the night-time HR and VT episodes in ICD recipients, with night-time HR of 50–70 bpm being the optimal therapeutics target. This shows the importance of a balanced beta-blocker pharmacotherapy in this group of patients [36].

### Limitations of the Study

Our research is fraught with certain limitations. To begin with, it was conducted at a single center, resulting in an underrepresentation of women and a restricted number of patients for the analysis, especially in the particular subgroups. This may limit the generalizability of the findings to other populations or healthcare settings. Secondly, the study’s retrospective design may introduce biases due to incomplete data in medical records and the lack of long-term outcome assessment, such as arrhythmia recurrence or mortality. The inducibility of ventricular arrhythmia on exertion was not tested. Moreover, there is a lack of official recommendations on whether a patient should or should not undergo coronary angiography after the high-energy ICD intervention transfers to more individually made decisions in terms of further diagnostic process. Therefore, the characteristics and management practices at this center may not be representative of those elsewhere. Addressing these limitations in future studies could strengthen the evidence base and provide a more comprehensive understanding of the factors influencing arrhythmia recurrence in ICD patients.

## 5. Conclusions

As we have mentioned in the “Limitations section”, the study’s retrospective design may introduce biases due to the lack of long-term outcome assessment, such as arrhythmia recurrence or mortality; however, we would like to stress the significance of in-hospital management and detection of patients with high likelihood of VT recurrence. Higher NYHA class and presence of left ventricular hypertrophy on TTE are independently associated with recurrent VT. Other factors heightening the risk of arrhythmia recurrence are history of chronic kidney disease and intensive insulin therapy, elevated WBC and serum creatinine levels, longer QTc in ECG, higher number of PVCs, and minimal heart rate in Holter monitoring. Clinicians need to actively search for the underlying cause of ventricular arrhythmia, which enables cessation of the trigger factor and optimization of heart failure treatment. This study corroborated a low referral rate for coronary angiography among patients with ICD discharge. There is a need to consider more frequent use of coronary angiography to assess significant coronary artery disease (CAD) in ICD recipients. Guidelines should endorse routine referral for coronary angiography in all patients admitted with ICD discharges. The study also indicates a significant association between CAD and the frequency of catheter ablation procedures in ICD patients, suggesting that CAD patients may benefit from more frequent catheter ablation interventions compared to those with non-ischemic etiology. Understanding these associations can guide personalized management strategies for ICD recipients.

## Figures and Tables

**Table 1:** Baseline characteristics of the study population and comparison of patients referred for coronary angiography or conservative management.

Variables		Overall Study Population n = 81	Coronary Angiography during In-Hospital Stay		*p*-Value
			No n = 47	Yes n = 34	
		n (%) or Mean ± SD			
Demographics and comorbidities					
Male		70 (86.4%)	39 (83.0%)	31 (91.2%)	0.288
Age [years]		63.6 ± 12.6	61.7 ± 15.0	66.3 ± 7.7	0.224
BMI [kg/m^2^]		28.4 ± 5.2	28.1 ± 5.0	28.8 ± 5.4	0.633
Current cigarette smoking		40 (50.0%)	20 (42.6%)	20 (60.6%)	0.112
NYHA		2.33 ± 0.7	2.3 ± 0.6	2.3 ± 0.8	0.780
CCS		1.4 ± 0.9	1.5 ± 1.1	1.3 ± 0.5	0.729
Arterial hypertension		43 (53.1%)	25 (53.2%)	18 (52.9%)	0.982
Diabetes mellitus		25 (30.9%)	10 (21.3%)	15 (44.1%)	0.028
Hyperlipidemia		64 (79.0%)	33 (70.2%)	31 (91.2%)	0.022
Chronic kidney disease		20 (25.0%)	13 (28.3%)	7 (20.6%)	0.433
History of AF		28 (34.6%)	19 (40.4%)	9 (26.5%)	0.500
History of MI		55 (67.9%)	30 (63.8%)	29 (87.9%)	0.016
Previous PCI/CABG	PCI	27 (34.2%)	10 (21.7%)	17 (51.5%)	<0.001
	CABG	9 (11.4%)	6 (13.0%)	3 (9.1%)	
	Both	18 (22.8%)	7 (15.2%)	11 (33.3%)	
History of ischemic stroke/TIA		12 (14.8%)	6 (12.8%)	6 (17.6%)	0.481
Hypertrophic cardiomyopathy		7 (8.6%)	4 (8.5%)	3 (8.8%)	0.645
Hyperthyroidism		16 (19.8%)	9 (19.2%)	7 (20.6%)	0.872
History of cardiac arrest		19 (23.5%)	12 (25.5%)	7 (20.6%)	0.604
Electrotherapy and management					
ICD implantation etiology	Ischemic	55 (67.9%)	25 (53.2%)	30 (88.2%)	0.001
	Non-ischemic	26 (32.1%)	22 (46.8%)	4 (11.8%)	
Implanted device	ICD	57 (70.4%)	33 (70.2%)	24 (70.6%)	0.971
	CRT-D	24 (29.6%)	14 (29.8%)	10 (29.4%)	
Appropriate intervention (VT)		77 (95.1%)	44 (93.6%)	33 (97.1%)	0.480
Inappropriate intervention	AF	3 (3.7%)	2 (4.3%)	1 (2.9%)	0.658
	Supraventricular tachycardia	1 (1.2%)	1 (2.1%)	0 (0.0%)	
	T-wave oversensing	1 (1.2%)	1 (2.1%)	0 (0.0%)	
Number of high-energy interventions		6.1 ± 6.9	4.1 ± 3.5	8.6 ± 9.0	0.086
Electrical storm		54 (71.1%)	30 (66.7%)	24 (77.4%)	0.310
Sustained ventricular tachycardia on admission		11 (15.5%)	7 (16.7%)	4 (13.8%)	0.742
AF on admission		9 (12.3%)	8 (19.1%)	1 (3.2%)	0.042
Recurrent discharge during hospitalization		16 (19.8%)	7 (14.9%)	9 (26.5%)	0.197
Recurrent VT during hospitalization		21 (26.3%)	10 (21.3%)	11 (33.3%)	0.228
High-energy interventions in the medical history		52 (68.4%)	27 (62.8%)	25 (75.8%)	0.228
PCI during hospitalization		8 (9.9%)	0 (0%)	8 (23.5%)	<0.001
Ablation due to VT during hospitalization		36 (45.0%)	20 (42.5%)	16 (47.1%)	0.750
Echocardiographic and electrocardiographic parameters					
LVEF [%]		29.5 ± 12.4	31.6 ± 13.9	26.5 ± 9.4	0.129
LAd [mm]		46.0 ± 7.2	46.1 ± 8.0	45.9 ± 5.9	0.945
LVEDV [mL]		221.5 ± 89.5	209.4 ± 93.6	236.7 ± 84.8	0.310
LVESV [mL]		149.3 ± 88.6	129.2 ± 88.3	177.0 ± 86.8	0.322
LV hypertrophy		31 (44.9%)	17 (43.6%)	14 (46.7%)	0.799
Holter monitoring during hospitalization	mean HR [bpm]	65.2 ± 9.9	66.4 ± 11.1	63.0 ± 7.1	0.363
	max HR [bpm]	88.8 ± 16.0	90.1 ± 17.9	86.8 ± 12.3	0.833
	min HR [bpm]	54.5 ± 10.0	55.5 ± 12.4	53.3 ± 5.7	0.841
	number of PVCs	4753.7 ± 10220.8	6734.6 ± 12464.4	1386.3 ± 2361.9	0.482
RBBB		16 (28.1%)	11 (30.6%)	5 (23.8%)	0.585
LBBB		7 (12.7%)	4 (11.8%)	3(14.3%)	0.785
QTc [ms]		453.1 ± 57.8	447.6 ± 64.2	465.2 ± 44.0	0.394
Laboratory tests					
Hgb [g/dL]		13.7 ± 1.8	13.7 ± 1.9	13.5 ± 1.8	0.670
WBC [1000/mm^3^]		9.0 ± 3.1	9.0 ± 3.3	9.0 ± 3.0	0.730
K+ [mmol/L]		4.4 ± 0.5	4.4 ± 0.5	4.4 ± 0.5	0.927
LDL [mg/dL]		80.9 ± 36.9	86.0 ± 42.5	74.6 ± 27.7	0.361
TCH [mg/dL]		146.2 ± 43.00	149.6 ± 49.1	141.9 ± 34.3	0.675
CRP [mg/L]		50.7 ± 100	61.5 ± 127.2	33.7 ± 28.3	0.497
hsTnT [ng/mL]		0.106 ± 0.16	0.044 ± 0.04	0.162 ± 0.21	0.093
TSH [uIU/mL]		2.0 ± 1.4	2.1 ± 1.3	1.9 ± 1.7	0.383
eGFR [ml/min/1.73 m^2^]		68.4 ± 18.8	68.4 ± 18.8	64.4 ± 21.0	0.467
sCr [mg/dL]		1.2 ± 0.6	1.1 ± 0.4	1.4 ± 0.9	0.433

SD—Standard deviation; BMI—Body mass index; NYHA—The New York Heart Association Classification; CCS—Canadian Cardiovascular Society grading of angina pectoris; =ICD—Implantable Cardioverter-Defibrillator; CRT-D—Implantable Cardiac Resynchronization Therapy defibrillator; VT—Ventricular Tachycardia; AF—Atrial fibrillation; PCI—Percutaneous Coronary Intervention; LV—Left Ventricle; LVEF—Left Ventricular Ejection fraction; LAd—Left Atrial diameter; LVEDV—Left Ventricular End Diastolic Volume; LVESV—Left Ventricular End-systolic volume; HR—Heart Rate; PVC—Premature Ventricular Contraction; RBBB—Right Bundle Branch Block; LBBB—Left Bundle Branch Block; QTc—corrected QT interval; MI—Myocardial Infarction; CABG—Coronary Artery Bypass Grafting; TIA—Transient Ischemic Attack; Hgb—Hemoglobin; WBC—White Blood Cell count; LDL—Low-Density Lipoprotein; TCH—Total Cholesterol; CRP—C-Reactive Protein; hsTnt—High-sensitivity troponin T; TSH—Thyroid Stimulating Hormone; eGFR—estimated Glomerular Filtration Rate; sCr—serum creatinine.

**Table 2:** Demographic and clinical characteristics in patients with and without recurrent VT during hospitalization.

		No Recurrent VT during Hospitalization (n = 60)	Recurrent VT during Hospitalization (n = 21)	*p*-Value
		n (%) or Mean ± SD	n (%) or Mean ± SD	
Demographics and comorbidities				
Male		53 (88.3%)	17 (81.0%)	0.395
Age [years]		62.3 ± 13.8	67.4 ± 7.5	0.217
BMI [kg/m^2^]		27.9 ± 5.4	29.7 ± 4.5	0.116
NYHA		2.2 ± 0.6	2.9 ± 0.8	0.028
CCS		1.4 ± 0.9	1.7 ± 1.2	0.775
Arterial hypertension		32 (53.3%)	11 (52.4%)	0.940
Diabetes mellitus		17 (28.3%)	8 (38.1%)	0.405
Insulin therapy		3 (5.0%)	5 (23.8%)	0.013
Chronic kidney disease		11 (18.6%)	9 (42.9%)	0.028
Current cigarette smoking		27 (45.8%)	13 (61.9%)	0.204
History of AF		22 (36.7%)	6 (33.3%)	0.796
History of MI		44 (74.6%)	15 (71.4%)	0.778
Previous PCI/CABG	PCI	20 (34.5%)	7 (33.3%)	0.669
	CABG	8 (13.8%)	1 (4.8%)	
	Both	12 (20.7%)	6 (28.6%)	
Ischemic stroke/TIA in medical history		8 (13.3%)	4 (19.0%)	0.591
History of cardiac arrest		14 (23.3%)	5 (23.8%)	0.965
Electrotherapy and management				
ICD implantation etiology	Ischemic	39 (65.0%)	16 (76.2%)	0.344
	Non-ischemic	21 (35.0%)	5 (23.8%)	
Implanted device	ICD	44 (73.3%)	13 (61.9%)	0.324
	CRT-D	16 (26.7%)	8 (38.1%)	
Appropriate intervention (VT)		56 (93.3%)	21 (100.0%)	0.225
Inappropriate intervention	AF	3 (5.0%)	0 (0%)	0.601
	Supraventricular tachycardia	1 (1.7%)	0 (0%)	
	T-wave oversensing	1 (1.7%)	0 (0%)	
Number of high-energy interventions		6.3 ± 7.4	5.1 ± 3.8	0.957
Electrical storm		38 (66.7%)	16 (84.2%)	0.144
Sustained ventricular tachycardia on admission		7 (12.7%)	4 (25.0%)	0.232
AF on admission		5 (9.3%)	4 (21.1%)	0.179
Recurrent discharge during hospitalization		5 (8.3%)	11 (52.4%)	<0.001
Number of recurrent discharges		2.5 ± 0.7	23.2 ± 43	0.310
High-energy interventions in the medical history		38 (65.5%)	14 (77.8%)	0.328
Coronary angiography during hospitalization		23 (38.3%)	11 (52.4%)	0.262
PCI during hospitalization		6 (16.2%)	2 (13.3%)	0.794
Ablation due to VT during hospitalization		20 (33.3%)	16 (80.0%)	0.001
Ablation site	LV	17 (89.5%)	14 (87.5%)	0.855
	RV	2 (10.5%)	2 (12.5%)	
Echocardiographic and electrocardiographic parameters				
LVEF [%]		31 ± 13.3	25.4 ± 8.4	0.093
LAd [mm]		45.7 ± 7.3	47.2 ± 7.0	0.734
LVEDV [mL]		220.3 ± 96.6	225.0 ± 70.4	0.978
LVESV [mL]		148.3 ± 94.8	155.0 ± 56.3	0.911
LV hypertrophy		19 (35.9%)	12 (75.0%)	0.006
Holter monitoring during hospitalization	mean HR [bpm]	64.0 ± 8.7	72 ± 13.7	0.160
	max HR [bpm]	87.0 ± 14.4	98.7 ± 21.2	0.128
	min HR [bpm]	52.3 ± 7.6	63.7 ± 13.6	0.033
	number of PVCs	1435.7 ± 2556.7	16367.0 ± 17578.1	0.012
RBBB		13 (28.9%)	3 (25.0%)	0.790
LBBB		5 (11.6%)	2 (16.7%)	0.643
QTc [ms]		433.4 ± 35.2	512.3 ± 77.0	0.028
Laboratory tests				
Hgb [g/dL]		13.9 ± 1.8	13.0 ± 1.9	0.099
WBC [1000/mm^3^]		8.5 ± 2.7	10.5 ± 3.8	0.018
K+ [mmol/L]		4.3 ± 0.5	4.6 ± 0.4	0.063
LDL [mg/dL]		82.7 ± 38.4	76.2 ± 32.8	0.503
TCH [mg/dL]		147.6 ± 43.4	142.2 ± 43.0	0.613
CRP [mg/L]		26.0 ± 18.6	75.4 ± 139.7	0.691
hsTnT [ng/mL]		0.1 ± 0.2	0.1 ± 0.1	0.440
TSH [uIU/mL]		2.0 ± 1.5	2.1 ± 1.4	0.778
eGFR [ml/min/1.73 m^2^]		70.7 ± 18.7	55.5 ± 18.8	0.002
sCr [mg/dL]		1.1 ± 0.4	1.5 ± 1.0	0.007

SD—Standard deviation; BMI—Body mass index; NYHA—The New York Heart Association Classification; CCS—Canadian Cardiovascular Society grading of angina pectoris; ICD—Implantable Cardioverter-Defibrillator; CRT-D—Implantable Cardiac Resynchronization Therapy defibrillator; VT—Ventricular Tachycardia; AF—Atrial fibrillation; PCI—Percutaneous Coronary Intervention; LV—Left Ventricle; RV—Right Ventricle, LVEF—Left Ventricular Ejection fraction; LAd—Left Atrial diameter; LVEDV—Left Ventricular End Diastolic Volume; HR—Heart Rate; PVC—Premature Ventricular Contraction; RBBB—Right Bundle Branch Block; LBBB—Left Bundle Branch Block; QTc—corrected QT interval; MI—Myocardial Infarction; CABG—Coronary Artery Bypass Grafting; TIA—Transient Ischemic Attack; Hgb—Hemoglobin; WBC—White Blood Cell count; LDL—Low-Density Lipoprotein; TCH—Total Cholesterol; CRP—C-Reactive Protein; hsTnt—High-sensitivity troponin T; TSH—Thyroid Stimulating Hormone; eGFR—estimated Glomerular Filtration Rate; sCr—serum Creatinine.

**Table 3:** Univariate and logistic regression analysis of different predictors of recurrent ventricular tachycardia during hospitalization.

Variable	Univariate Analysis			Logistic Regression Analysis AUC = 0.853. 95%CI 0.716 to 0.941; Hosmer–Lemeshow *p* = 0.57		
	OR	95%CI	*p*-Value	OR	95%CI	*p*-Value
Non-Ischemic etiology	0.58	0.19–1.81	0.348	-	-	-
PCI	0.63	0.10–3.78	0.613	-	-	-
QTc (per 1 ms)	1.03	0.99–1.08	0.09	-	-	-
NYHA (per 1 class)	4.74	1.29–17.44	0.019	4.63	1.07–19.99	0.040
LVH	5.37	1.52–18.99	0.009	10.59	1.01–111.26	0.049
LVEF (per 1%)	0.96	0.91–1.01	0.086	-	-	-
Insulin therapy	5.94	1.28–27.56	0.023	-	-	-
WBC (per 1000/mm^3^)	1.23	1.04–1.45	0.016	-	-	-
eGFR	0.96	0.93–0.99	0.005	-	-	-
hsTnT (per 1 ng/mL)	0.82	0.01–98.59	0.936	-	-	-

NYHA—New York Heart Association classification; LVH—left ventricular hypertrophy; LVEF—left ventricular ejection fraction; WBC—white blood cells; eGFR—estimated glomerular filtration rate; hsTnT—high sensitivity troponin T.

## Data Availability

The raw data supporting the conclusions of this article will be made available on request to the corresponding author.
